# The protective role of lutein on isoproterenol-induced cardiac failure rat model through improving cardiac morphology, antioxidant status via positively regulating Nrf2/HO-1 signalling pathway

**DOI:** 10.1080/13880209.2019.1649436

**Published:** 2019-08-14

**Authors:** Bo Ouyang, Zili Li, Xiongying Ji, Jiangwei Huang, Hengsheng Zhang, Changrong Jiang

**Affiliations:** a Department of Traditional Chinese Medicine Rehabilitation, Affiliated Nanhua Hospital, University of South China, Hengyang, China;; b Department of Cardiology, Affiliated Nanhua Hospital, University of South China, Hengyang, China;; c Department of Gastroenterology, Affiliated Nanhua Hospital, University of South China, Hengyang, China

**Keywords:** Myocardial infarction, infarct size, cardioprotective, cardiac markers

## Abstract

**Context:** Lutein (LU) is a major carotenoid with various pharmacological activities including anti-inflammatory, antioxidant and anti-apoptosis.

**Objective:** The cardioprotective efficacy of LU was determined by evaluating the biochemical and histopathological changes in isoproterenol (ISO) induced myocardial infarction (MI) rat model.

**Materials and methods:** Healthy male albino rats (*n* = 40) were segregated into 4 equal groups. Group I (control) rats were administered with olive oil, Group II (LU) rats were orally pre-treated with only 40 mg of LU for 28 days, Group III (MI induced) rats were injected (subcutaneously; s.c) with 85 mg/kg of ISO for 2 consecutive days, whereas Group IV (LU + ISO) rats were pre-treated with 40 mg of LU for 28 days before ISO induction.

**Results:** ISO-induced group showed increased infarct size and cardiac/inflammatory/apoptotic markers. However, pre-treatment with LU (28 days) considerably reduced (*p* < 0.01) the infarct size (14%), lipid peroxidation product (MDA;42%), cardiac markers [(lactate dehydrogenase (LDH) and creatine kinase-MB (CK-MB), cardiac troponin T (cTn T)], inflammatory markers [IL-1β, IL-6, tumour necrosis factor alpha (TNF-α), nuclear factor kappa B p65 subunit (NF-κB p65)] and apoptotic markers (caspase-3 and -9). Also, LU significantly improved (*p* < 0.01) the antioxidants [catalase (CAT), superoxide dismutase (SOD)] as well as markedly upregulated (*p* < 0.01) the protein expression of HO-1 and Nrf2. Moreover, LU considerably reversed all the histopathological changes and thus exhibits its cardioprotective activity.

**Conclusion:** LU exhibits potent cardioprotective activity against ISO-induced cardiotoxicity and might be recommended with standard cardioprotective agents for treating various MI-related complications.

## Introduction

Heart attack or myocardial infarction (MI) is one of the deadliest forms of ischaemic heart disease as it results in high mortality and morbidity. Recently, China Heart Failure Symposium/Association and World Health Organization (WHO) reported that approximately 50% of total cardiovascular disease (CVD)-related mortality is due to MI (Li et al. 2015a). MI is caused by insufficient oxygenated blood flow to the myocardium (via a coronary artery) which results in an imbalance in oxygen/nutrient demand and eventually leads myocardial damage or injury (Amran et al. 2015). Many scientists have demonstrated various pathophysiological and biochemical events including oxidative stress (lipid peroxidation), inflammatory response, necrosis, apoptosis, hyperlipidaemia, etc. could contribute to MI (Reed et al. 2017; Lu et al. 2018). However, to date, the exact reason or pathophysiology behind MI is still obscure. Moreover, the current treatment regimen for MI (anti-thrombotic/anti-coagulant) is very limited as it triggers various serious adverse effects including heartburn, hypertension, gastrointestinal disorders. Therefore, there is a quest for novel natural cardio-therapeutic agents with potent antioxidant, anti-inflammatory and anti-apoptotic properties that would limit or protect myocardial injury or damage with no or fewer adverse effects (Upaganlawar et al. 2011; Wong et al. 2017).

Lutein (LU) is a major xanthophyll pigment (carotenoid-C_40_ H_56_ O_2_) commonly present in eggs, dark green or yellow-coloured vegetables and fruits such as kale, spinach, carrot, celery, kiwi, marigold, avocado and broccoli (Nwachukwu et al. 2016). LU is highly recommended for ocular health (functional food) as it accumulates in macula lutea and protects the retina from oxidative damage (Richer et al. 2007). LU possesses numerous pharmacological activities including anti-inflammatory, antidiabetic, antioxidant, anti-apoptotic and anticancer (Wu et al. 2015; Nwachukwu et al. 2016; Hwang et al. 2018) as well as neuroprotective, renoprotective, osteoprotective and hepatoprotective activities (Li et al. 2015b; Qiao et al. 2018). Previously, astaxanthin, a red-pigmented carotenoid (similar structure as LU except for keto group) has been reported to display cardioprotective activity against ISO-induced cardiotoxicity in a rat model (Hussein 2015). Moreover, a commercial antioxidant mix (VitaePro) rich in LU, astaxanthin and zeaxanthin is reported to protect myocardium in a rat *ex vivo* ischaemic/reperfusion injury model (Adluri et al. 2013).

Ample amount of studies indicated that supramaximal doses of ISO can trigger overproduction of free radicals which eventually modulate redox signalling pathway like nuclear factor erythroid 2-related factor 2 (Nrf2)/heme oxygenase 1 (HO-1) to protect myocardium from free radical (oxidative stress) induced damage or injury by enhancing oxidative status (Sahu et al. 2015; Li et al. 2016a). Hence, for the current study, the protein expression of transcriptional factors involved in the Nrf2/HO-1 redox signalling pathway including Nrf2 and HO-1 were explored. Based on the various biological properties of LU, we speculate that LU might show cardioprotective property against isoproterenol (ISO)-induced cardiotoxicity due to its potent antioxidant (via altering Nrf2/HO-1 redox signalling pathway), anti-apoptotic and anti-inflammatory activities. Hence, the current animal experiment was designed to explore the cardioprotective efficacy of LU by evaluating the infarct size, cardiac markers, antioxidant status, inflammatory markers, apoptotic markers and histopathological changes in MI rat model.

## Materials and methods

### Chemicals and reagents

LU (98% pure HPLC grade), ISO, formalin, triphenyl tetrazolium chloride (TTC), RIPA buffer and xylene were purchased from Sigma-Aldrich (Burlington, MA, USA). Other chemicals and reagents used in this study are of either analytical or HPLC grade.

### Experimental rats

Healthy albino Sprague-Dawley male rats of total 40 (*n* = 40) weighing 215 ± 10 g were purchased and maintained under standard laboratory conditions (21 ± 2 °C; 60–65% humidity) at 14/10 h light and dark cycle in a polycarbonate cage. All the animal experimental procedures and protocols used in this study are approved by the ethical committee board of the University of South China (ECB-USC-10/234-13). Similarly, all the animals were handled based on the guidelines formulated by the National Institute of Health (NIH) containing Guide for the care and handling of laboratory animals.

### Experimental grouping

All the healthy rats were divided into 4 equal groups. Group I (control; *n* = 10) rats were administered with olive oil, Group II (LU; *n* = 10) rats were pre-treated (orally; p.o) with only 40 mg of LU (dissolving with olive oil) for 28 days, Group III (MI induced; ISO *n* = 10) rats were injected (s.c) with 85 mg/kg of ISO for 2 consecutive days (29th and 30th day) (Amran et al. 2015; Hassan et al. 2015). Group IV (LU + ISO; *n* = 10) rats were pre-treated with 40 mg of LU for 28 days before ISO exposure.

### Sample collection and processing

At the end of the experimental period (31st day), all the overnight fasted rats were weighed using standard laboratory animal weighing scale. Rats were euthanized (NIH guidelines) under a high dose of pentobarbital sodium (55 mg/kg b.wt) and the blood sample was immediately collected. Cardiac tissue (heart) was excised immediately from all the sacrificed rats and weighed using a standard laboratory animal weighing scale. A small portion of cardiac tissue was fixed in 10% formalin for histopathological analysis and for TTC staining procedure. The remaining cardiac tissue was homogenized (10%) using 0.1 M Tris-HCl buffered solution and centrifuged at 12,000 *g* for 12 min at 4 °C and the resulted supernatant was stored at −80 °C until analysis. From the collected blood sample, the serum was extracted by centrifuging the blood at 3500 *g* for 10 min at 4 °C.

### Infarct size

The cardiac infarct size was determined by TTC staining procedure based on the method of Panda et al. (2014). Briefly, the transverse cardiac slice (mid-ventricular-base apex) of 1–2 mm thickness and probed (incubate) with 1% TTC stain for 40 min at 37 °C and followed by fixing with 10% formaldehyde. The normal cardiac tissue (non-infarct region) was appeared in red, whereas abnormal cardiac tissue (infarct region) has appeared in pale grey or white. A digital camera was used to photograph the cardiac section and the infarct size was measured using Image-Pro software (Ver. 10) from Media Cybernectics, Inc. (Rockville, MD, USA).

### Antioxidant status

Cardiac homogenate (supernatant) was used to assess the levels of malondialdehyde (MDA-lipid peroxidation product) by an enzymatic assay kit brought from Kangchen Biotechnology (Shanghai, China) based on the manufacturer’s instruction. However, the activities of endogenous cardiac antioxidants like catalase (CAT) and superoxide dismutase (SOD) were measured using the enzymatic assay kit brought from Cayman Chemical (Ann Arbor, MI, USA) based on manufacturer’s protocol.

### Cardiac markers

The activities of serum diagnostic cardiac marker enzymes like lactate dehydrogenase (LDH) and creatine kinase-MB (CK-MB) were determined using commercial ELISA kit purchased from Teco Diagnostics (Anaheim, CA, USA) and cardiac troponin T (cTn T) was measured by ELISA assay kit bought from MyBioSource (San Diego, CA, USA).

### Inflammation markers

The nuclear/cytosolic fractionation kit was bought from BioVision Inc. (Milpitas, CA, USA) to extract the cytosolic and nuclear fraction from the heart tissue homogenate (supernatant) using ultracentrifugation technique. Then the levels of various cardiac inflammatory markers like IL-1β, IL-6, TNF-α were measured in cytosolic fraction using commercial rat specific ELISA kit (Ray Biotechnology, Inc., Minneapolis, MN, USA). Meanwhile, the levels of NF-κB p65, an active NF-κB subunit was measure in cardiac nuclear fraction using from ELISA NF-κB p65 transcription factor assay kit from Abcam (Cambridge, UK).

### Apoptotic markers

Both caspase-3 and -9 (major apoptotic markers) are assessed in cardiac tissue homogenate by an enzymatic ELISA kit bought from MyBioSource (San Diego, CA, USA) based on the manufacturer’s procedure.

### Protein expression by Western blot

The Western blot technique was employed to quantify the protein expression of Nrf2 (nuclear fraction) and HO-1 (cytosolic fraction) of cardiac homogenate. The protein levels were estimated using a BCA protein assay kit from BioVision Inc. (Milpitas, CA, USA) by treating cardiac tissue homogenate (supernatant) with lytic RIPA buffered solution containing various proteinase. Protein (40 µg) was resolved using 10% SDS-PAGE apparatus (from each group) and electrotransferred onto polyvinylidene difluoride (PVDF) membrane. Then the membrane was blocked with Tween 20, 5% skimmed milk and PBS solution and probed with primary antibodies (rabbit polyclonal anti-Nrf2 antibody (1:800); rabbit polyclonal anti-HO-1 antibody (1:1200); and housekeeping anti-β actin (1:1000); anti-histone H3 antibody (1:1000) were bought from Abcam, Cambridge, UK) for overnight at 4 °C, followed by exposing to secondary antibody-rabbit polyclonal anti-horseradish peroxidase (HRP) antibody (1:10,000 from Abcam, Cambridge, UK) and incubated for 1 h at 37 °C. The protein bands in the PVDF membrane was developed using enhanced chemiluminescence (ECL) kit and the protein expression was measured using image analyzing software (Image-Pro software; Ver. 10) from the Media Cybernectics, Inc. (Rockville, MD, USA).

### Histopathological analysis

A section of heart tissue was fixed in 10% formalin solution and embedded in paraffin wax to form a tissue block. Then the block was cut into 4 mm slice using ultra-microtome and stained with haematoxylin and eosin (H&E) stain and viewed under a light microscope (Olympus DP72, Tokyo, Japan) and photographed using a digital camera. The cardiac morphological changes in each rat section were analyzed by the pathologist (blinded from the group and experimental study) with the help of Image-Pro software; Ver. 10) from the Media Cybernectics, Inc. (Rockville, MD, USA).

### Statistical analysis

Values are explicated as the mean ± standard error of mean (SEM). The significant (^#^*p* < 0.01, *p < 0.05) difference between the experimental group (ISO vs. Control group; LU + ISO vs. ISO group) was determined using GraphPad Prism (Version:6; GraphPad Software Inc., La Jolla, San Jose, CA, USA) by one-way ANOVA followed by Tukey’s *post hoc* multi-comparison test with a *p*-value less than 0.05 is deemed as significant.

## Results

### Effect of LU on heart weight, body weight and heart to body weight ratio

A pronounced increase (*p* < 0.01) in heart weight, as well as the heart to body weight ratio, were noted in ISO-induced MI rat group as compared with control rats (Table 1). Rats pre-treated with LU for 28 days prior ISO exposure (LU + ISO) would significantly lower (*p* < 0.01) the heart weight (from 0.825 to 0.672 g) as well as the heart to body weight ratio (from 0.379 to 0.304%) than ISO-induced rats. There is no significant difference in the case of body weight in any of the experimental groups.

**Table 1.** Effect of LU on changes in body weight, heart weight and heart to body weight ratio.

	Control	LU	ISO	LU + ISO
Heart weight (g)	0.610 ± 0.04	0.605 ± 0.04	0.825 ± 0.06a^#^	0.672 ± 0.05b^#^
Body weight (g)	219.00 ± 7.00	220.50 ± 6.00	217.50 ± 5.00a^NS^	221.00 ± 7.00b^NS^
Heart to body weight ratio (%)	0.278 ± 0.01	0.274 ± 0.01	0.379 ± 0.02a^#^	0.304 ± 0.01b^#^

Values are presented as the mean ± standard error of mean (SEM). The probability value (*p*-value; ^#^*p* < 0.01, *p < 0.05): where ‘a’ indicates the significant difference between ISO group and Control group, while ‘b’ indicates the significant difference between LU + ISO group and ISO induced groups. LU: lutein; ISO: isoproterenol; NS: non-significant.

### Effect of LU on cardiac infarct size

**Figure 1.**
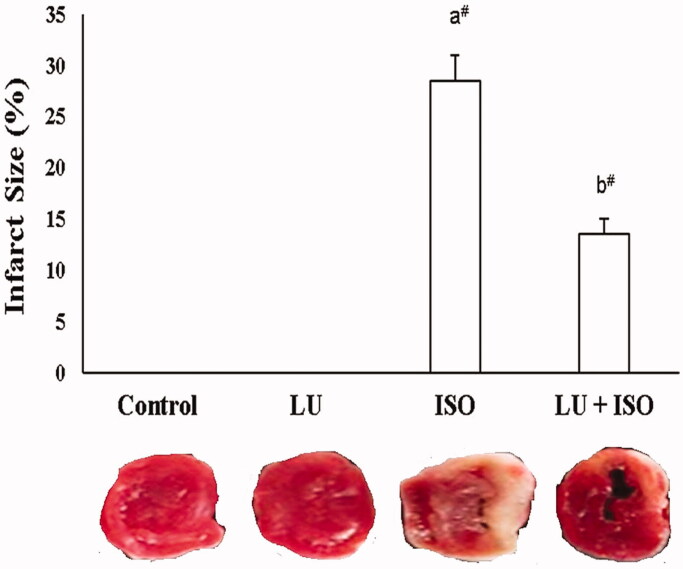
Effect of LU on cardiac infarct size. Values are explicated as the mean ± standard error of mean (SEM). The probability value (*p*-value; ^#^*p* < 0.01, *p < 0.05): where ‘a’ indicates the significant difference between the ISO group and the Control group, while ‘b’ indicates the significant difference between LU + ISO group and ISO-induced groups. LU: lutein; ISO: isoproterenol.

Figure 1 depicts the change in cardiac infarct size in experimental animals. The infarct size of ISO-induced rats was substantially elevated (*p* < 0.01; 29%) in comparison with control rats. Upon, administration with LU for 28 days prior to ISO induction showed a marked decrease (*p* < 0.01; 14%) in the infarct size in comparison with ISO-induced MI model group.

### Effect of LU on the cardiac lipid peroxidation product and antioxidant enzymes

The average value of lipid peroxidation product (like MDA) was significantly increased (*p* < 0.01; 56%), and the activities of various cardiac antioxidants like CAT and SOD were significantly decreased (*p* < 0.01) in ISO-induced rat (Table 2). However, rats supplemented with 40 mg of LU for 28 days showed a substantial reduction (*p* < 0.01) in lipid peroxidation products like MDA (42%) along with marked improvement (*p* < 0.01) in the activities of cardiac antioxidants like CAT and SOD than ISO alone injected rats.

**Table 2.** Effect of LU on the heart lipid peroxidation products and antioxidant enzymes.

Parameters	Control	LU	ISO	LU + ISO
MDA (nmol/mg protein)	0.59 ± 0.06	0.61 ± 0.08	1.35 ± 0.15a^#^	0.78 ± 0.08b^#^
SOD (U/mg protein)	5.05 ± 0.50	5.03 ± 0.50	3.31 ± 0.33a^#^	4.52 ± 0.47b^#^
CAT (U/mg protein)	14.84 ± 1.40	14.90 ± 1.60	10.80 ± 1.10a^#^	12.22 ± 1.20b*

Values are presented as the mean ± standard error of mean (SEM). The probability value (*p*-value; ^#^*p* < 0.01, *p < 0.05): where ‘a’ indicates the significant difference between ISO group and Control group, while ‘b’ indicates the significant difference between LU + ISO group and ISO induced groups. LU: lutein; ISO: isoproterenol; NS: non-significant.

### Effect of LU on serum cardiac markers enzymes

Table 3 represents the activities of serum cardiac diagnostic marker enzymes like cTn T, CK-MB and LDH in experimental rats. The activities of serum cardiac diagnostic marker enzymes like cTn T, CK-MB and LDH were greatly elevated (*p* < 0.01) in MI-induced (ISO) group vs. control group. In contrast, those elevated cardiac diagnostic marker enzymes (cTn T, CK-MB and LDH) were dramatically lowered (*p* < 0.01) in LU-pre-treated rats as compared with ISO-exposed rats.

**Table 3.** Effect of LU on the serum cardiac diagnostic markers enzymes.

Parameters	Control	LU	ISO	LU + ISO
cTn T (ng/mL)	0.52 ± 0.04	0.55 ± 0.05	1.27 ± 0.11a^#^	0.75 ± 0.07b^#^
CK-MB (IU/L)	71.00 ± 9.10	69.80 ± 8.50	151.00 ± 14.00a^#^	88.00 ± 9.00b^#^
LDH (IU/L)	86.90 ± 11.20	89.30 ± 10.40	165.30 ± 15.00a^#^	106.60 ± 11.00b^#^

Values are presented as the mean ± standard error of mean (SEM). The probability value (*p*-value; ^#^*p* < 0.01, *p < 0.05): where ‘a’ indicates the significant difference between ISO group and Control group, while ‘b’ indicates the significant difference between LU + ISO group and ISO induced groups. LU: lutein; ISO: isoproterenol; NS: non-significant.

### Effect of LU on cardiac inflammatory markers

**Figure 2.**
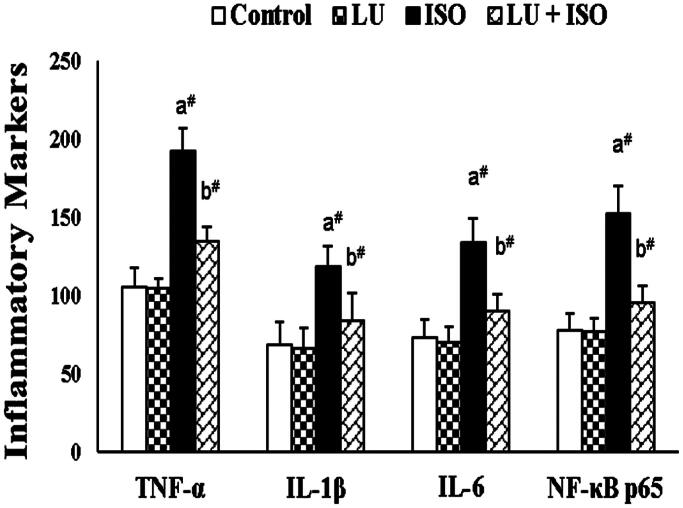
Effect of LU on cardiac inflammatory markers. Values are presented as the mean ± standard error of mean (SEM). The probability value (*p*-value; ^#^*p* < 0.01, *p < 0.05): where ‘a’ indicates the significant difference between the ISO group and the Control group, while ‘b’ indicates the significant difference between LU + ISO group and ISO-induced groups. LU: lutein; ISO: isoproterenol; IL-1β/6: interleukin one beta/six; TNF-α: tumour necrosis factor alpha; NF-κB: p65 subunit-nuclear factor kappa B p65 subunit.

As shown in Figure 2, the concentration of various pro-inflammatory cytokines like IL-1β, TNF-α, NF-κB p65 subunit and IL-6 in heart tissue homogenate were significantly inclined (*p* < 0.01) in rats exposed with ISO (MI model) than control rats. Twenty-eight days of treatment with LU (40 mg) before ISO injection would substantially decline (*p* < 0.01) the concentration of IL-1β, TNF-α, NF-κB p65 subunit and IL-6.

### Effect of LU on the apoptotic markers

**Figure 3.**
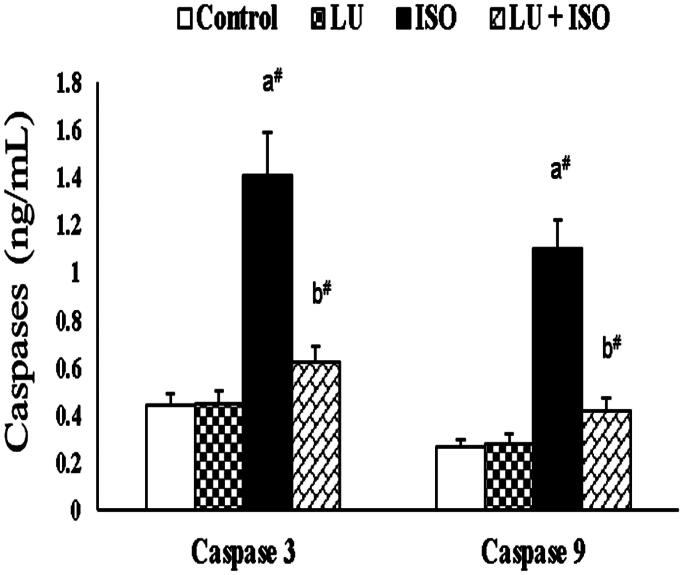
Effect of LU on cardiac apoptotic markers. Values are presented as the mean ± standard error of mean (SEM). The probability value (*p*-value; ^#^*p* < 0.01, *p < 0.05): where ‘a’ indicates the significant difference between the ISO group and the Control group, while ‘b’ indicates the significant difference between LU + ISO group and ISO-induced groups. LU: lutein; ISO: isoproterenol.

Figure 3 represents the effect of LU on cardiac apoptotic markers in experimental rats. An exponential increment (*p* < 0.01) in the levels of caspase-3 (68% increase), caspase-9 (75% increase) were observed in rats administered with ISO. However, the levels of caspase-3 (56% decrease), caspase-9 (61% decrease) were found to be statistically decreased (*p* < 0.01) in rats administered with LU for 28 days as pre-treatment regimen.

### Effect of LU on the protein expression of Nrf2 and HO-1

**Figure 4.**
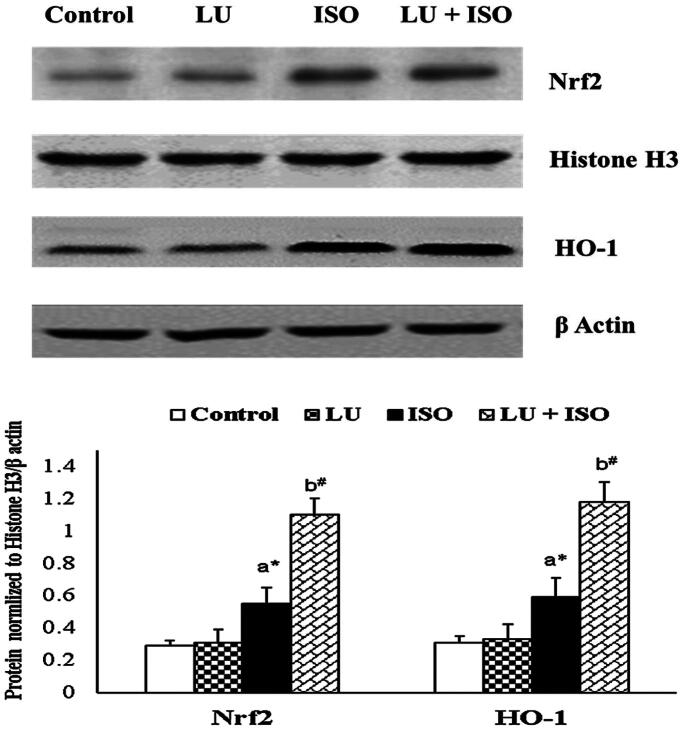
Effect of LU on protein expression of Nrf2 and HO-1. Values are presented as the mean ± standard error of mean (SEM). The probability value (*p*-value; ^#^*p* < 0.01, *p < 0.05): where ‘a’ indicates the significant difference between the ISO group and the Control group, while ‘b’ indicates the significant difference between LU + ISO group and ISO-induced groups. LU: lutein; ISO: isoproterenol; Nrf2: nuclear factor erythroid 2-related factor 2, HO-1: heme oxygenase.

The protein expression of Nrf2 and HO-1 in experimental rats was displayed in Figure 4. The protein expression of Nrf2 (nuclear fraction) and HO-1 (cytosolic fraction) were significantly upregulated (*p* < 0.05) in rats induced with ISO. On comparison with ISO-induced, the protein expression of Nrf2 (nuclear fraction) and HO-1 (cytosolic fraction) was furthermore upregulated (*p* < 0.01) in LU + ISO group rats.

### Effect of LU on the cardiac histopathological changes

**Figure 5.**
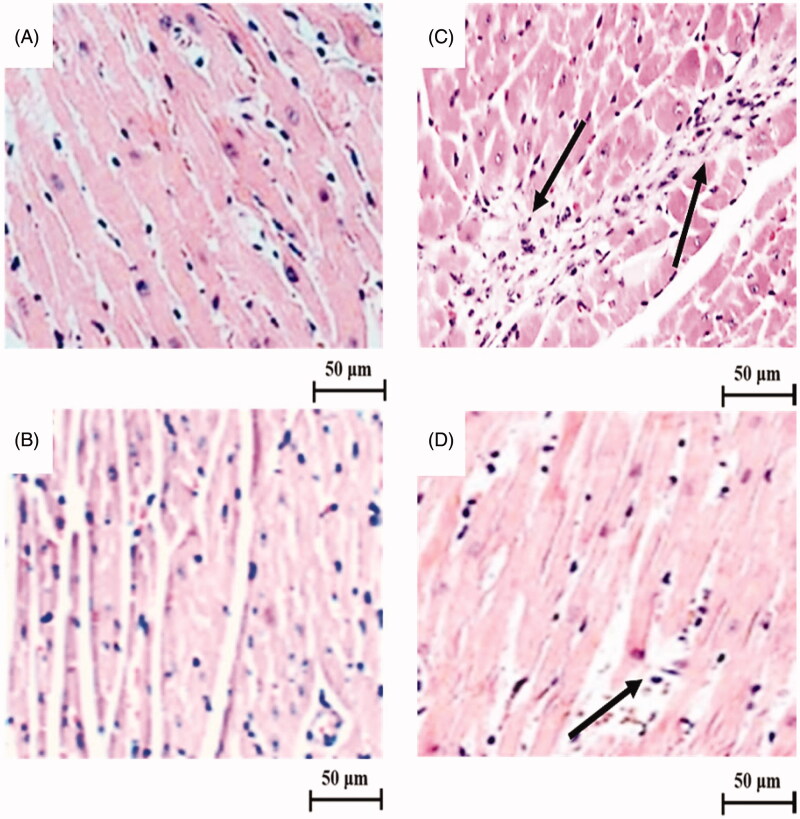
Effect of LU on cardiac histological changes under a light microscope (400×). The cardiac section of control rats (A) and LU alone rats (B) showed normal cardiac architecture with a proper myofibrillar arrangement without any abnormal histopathological changes. While the cardiac section of ISO-induced rats (C) depict disoriented myofibril with intense neutrophil infiltration (arrow mark) and many necrotic changes. The cardiac section of rats pre-treated with LU (D) before ISO-induced display mild neutrophil infiltration (arrow mark) and lesser necrotic changes with a better myofibrillar arrangement. Scale bar: 50 µm.

Figure 5 represents the effect of LU on cardiac histological changes (400×) in experimental rats. The cardiac section of control rats (Figure 5(A)) and LU alone (Figure 5(B)) treated rats showed normal cardiac architecture with a proper myofibrillar arrangement. While a cardiac section of ISO-induced rats (Figure 5(C)) depict disoriented myofibril with intense neutrophil infiltration (crowding pattern) and many necrotic changes. However, the cardiac section of rats pre-treated with LU (Figure 5(D)) before ISO-induced display mild neutrophil infiltration and lesser necrotic changes with the better myofibrillar arrangement.

## Discussion

ISO is a synthetic catecholamine which can elicit excessive stress (at supramaximal dose – 85 mg/kg) to cardiac tissue through excessive production of free radicals like ROS (auto-oxidation) which resulted in cardiac contractile dysfunction and myocardial damage (elevated lipid peroxidation products). Hence, ISO-induced MI is one of the popular non-invasive models to access the cardioprotective activity of any pharmacological agent (Li et al. 2016b; Khan et al. 2018). Results of this animal experiment suggest that LU pre-treatment could significantly reduce the infarct size, cardiac markers, inflammatory markers, lipid peroxidation product and apoptotic markers as well as significantly upregulated the protein expression of Nrf2 and HO-1 to endorse its strong cardioprotective activity against ISO-induced myocardial damage.

The major hallmark of MI is the large size of cardiac infarct area, which was measured using the TTC staining process (Panda et al. 2014). The size of the infarct region is larger in ISO-induced rats due to elevated myocardial damage or injury (insufficient blood supply/excessive oxidative stress). However, rats pre-treated with LU before ISO induction could significantly reverse the oxidative stress (antioxidant and anti-lipid peroxidation) and thereby protect the cardiac tissue from further damage or injury. Previously, Li et al. (2012) confirmed that treatment with LU considerably reduced the infarct size in the cerebral ischaemic/reperfusion mouse model.

As mentioned earlier, various pathophysiological and biochemical events including oxidative stress (lipid peroxidation), inflammatory response, necrosis, apoptosis were reported as the main contributor for MI (Reed et al. 2017; Lu et al. 2018) and hence for the current study, the antioxidant status, inflammatory and apoptotic markers are measured. In addition, myocardiocytes/myocytes are highly vulnerable to free radical damage owing to the high content of polyunsaturated fatty acids and high energy (ATP) demand. The level of MDA was exponentially increased along with the declined activities of various cardiac antioxidants (CAT and SOD) in ISO-injected rat due to increased free radical generation and subsequent myocardiocyte lipid peroxidation. In contrary, rats supplemented with LU for 28 days could considerably improve the antioxidant status (encounter excess free radicals) and lower MDA production by thus improving myocardiocytes integrity through suppressing lipid peroxidation. Similarly, Nidhi et al. (2015) confirmed that supplementation with LU and its metabolite could considerably improve the antioxidant activities of CAT, SOD in a rat model. Moreover, the free hydroxyl group (OH^−^) and the presence of several double bonds in LU might aid in scavenge the free radicals and thereby prevent the lipid peroxidation (Peng et al. 2016).

Evaluation of cardiac diagnostic marker enzymes during MI-related cardiotoxicity is a mandatory procedure. During this study, the rats injected with ISO (mimic MI model) showed substantial incline in the levels of various cardiac marker enzymes like cTn T, CK-MB, LDH. Due to elevated lipid peroxidation (oxidative stress) in cardiomyocytes and results in the leakage of cardiac enzymes from cardiac tissue into extracellular fluid serum (Li et al. 2016b). However, rats treated with 40 g of LU showed declined levels of marker enzymes like cTn T, CK-MB, LDH owing to membrane protective or anti-lipid peroxidation property (Nwachukwu et al. 2016; Qiao et al. 2018). Likewise, ISO-induced rats supplemented with a carotenoid (astaxanthin) demonstrated the decreased activity of various cardiac markers like CK-MB, LDH due to potent antioxidant and anti-lipid peroxidation properties.

Several reports have indicated that oxidative stress and inflammatory response are interconnected with each other (vicious cycle) as they both play a crucial inter-role in the induction of MI (Kumar et al. 2016; Reed et al. 2017). In the current study, the concentration of various inflammatory markers (pro-inflammatory cytokines) is substantially elevated in the ISO-induced group owing to the activation of NF-κB signalling cascade via oxidative stress. While pre-treated rats with LU before ISO injection would substantially decline the concentration of IL-1β, TNF-α, NF-κB p65 subunit and IL-6 by inhibiting the activation of the NF-κB signalling pathway. Our results are in correspondence with the outcome of Kim et al. (2011) as well as Wu et al. (2015). They both demonstrated that LU treatment could significantly inhibits free radical generation and thus suppress the activation of NF-κB and ultimately results in downregulation of various proinflammatory cytokines like TNF-α, IL-1β, IL-6. During ISO induction, various pro-apoptotic proteins are upregulated through the caspase cascade system (particularly caspase-3 and -9) to exhibit myocardiocyte apoptosis (Guo et al. 2013). Even in this study, we found that the levels of apoptotic markers like caspase-3 and -9 are considerably increased in ISO administered rats. Nonetheless, those apoptotic markers like caspase-3 and -9 were significantly reversed to normal level in LU supplemented group (LU-ISO). The above results are in agreement with the outcome of Nataraj et al. (2016), who also concluded that treatment with LU could considerably abolish the activity of caspase-3 and -9 in the mouse model.

Nrf2 is a crucial nuclear transcriptional factor which plays a major role in the regulation of redox homeostasis by inducing various antioxidant and detoxifying enzyme. Under normal physiological conditions, the Nrf2 form a complex with its negative regulator-Kelch-like ECM associated protein (Keap1) as an inactive form (Nrf2–Keap1 complex reside in the cytoplasm) and subsequently degraded by the various proteasome upon stimulation (Lee et al. 2014). But, during pathological or oxidative stress (ISO induction), the Nrf2 broke down (liberated-active form) from the Nrf2–Keap1 complex and translocate into nucleus (from cytoplasm) and bound to antioxidant response element (ARE) regions and subsequently modulate the expression of various antioxidant and detoxifying enzyme genes such as heme oxygenase 1 (HO-1), glutathione, NADPH:quinone oxidoreductase 1 (NQO-1) to protect the cells from further damage (Cheng et al. 2015).

As mentioned earlier, many reports are documented that supramaximal doses of ISO can trigger oxidative stress which ultimately modulates Nrf2/HO-1 signalling pathway to protect myocardium from oxidative stress by enhancing oxidative status (Sahu et al. 2015; Li et al. 2016a). Hence, for the current study, the protein expression of various transcriptional factors involved in Nrf2/HO-1 redox signalling pathway are explored. A marked upregulation in the protein expression of Nrf2 and HO-1 were observed in rats induced with ISO as a defense mechanism to lower the oxidative stress. But, the protein expressions of Nrf2 and HO-1 were found to be furthermore upregulated in rats pre-treated with LU. The activation of Nrf2 by LU might be due to the presence of electrophilic group which reacts with thiols of cysteine residues in Keap1 and trigger the breakdown of Nrf2–Keap1 complex (Wu et al. 2015; Li et al. 2015b). Therefore, we confirmed that LU can positively regulate the Nrf2/HO-1 signalling pathway and thus enhance the expression of various antioxidant and cytoprotective proteins and thereby endorsing its cardioprotective activity.

Those above-mentioned biochemical, molecular changes during ISO-induced MI are needed to be validated by histopathological analysis. The cardiac section of control rats and LU alone treated rats showed normal cardiac architecture with a proper myofibrillar arrangement. While the cardiac section of ISO-induced rats depicts disoriented myofibril (degeneration) with intense (large) neutrophil infiltration and many necrotic changes. The cardiac section of rats pre-treated with LU before ISO-induced revealed the mild neutrophil infiltration (small) and lesser necrotic changes with better myofibrillar arrangement due to the potent antioxidant, anti-inflammatory and anti-apoptotic activity. Likewise, the cardiac section of LU administered diabetic rats also showed fewer intercalated disk and necrotic changes owing to antioxidant and anti-lipid peroxidation properties (Sharavana et al. 2017). This experiment has few limitations like avoidance of necrosis and apoptotic analysis as well as heart rate/electrocardiogram measurement. However, this study has many positives such as quantification of protein expression of Nrf2/HO-1 to reveal the underpinning cardioprotective mechanism of LU with well supported by antioxidant, anti-inflammatory and anti-apoptotic assessment along with histo-morphological analysis.

## Conclusion

Overall, pre-treatment with 40 mg of LU could significantly reduce the cardiac infarct size, serum diagnostic cardiac markers, lipid peroxidation product (MDA), inflammatory markers (pro-inflammatory cytokines) and apoptotic markers (caspases) as well as markedly upregulated the protein expression of Nrf2 and HO-1 to confer its strong cardioprotective activity against ISO-induced myocardial damage. However, further clinical trials are needed to validate our results.
